# Assessing the role of dryness and burning sensation in diagnosing laryngopharyngeal reflux

**DOI:** 10.1038/s41598-024-55420-y

**Published:** 2024-02-24

**Authors:** Xiaowei Zheng, Zhiwei Chen, Ting Chen, Liqun Zhou, Chaofeng Liu, Jingyi Zheng, Renyou Hu

**Affiliations:** 1 Department of Otorhinolaryngology Head and Neck Surgery, Shengli Clinical Medical College of Fujian Medical University, 134 Dong Jie, Gulou District, Fuzhou City, 350001 Fujian Province China; 2 Chongqing Jinshan Science and Technology (Group) Co Ltd, Chongqing, 401120 China

**Keywords:** Epidemiology, Gastrointestinal diseases, Digestive signs and symptoms, Diseases

## Abstract

Laryngopharyngeal reflux disease (LPRD) is a condition characterized by the regurgitation of stomach and duodenal contents into the laryngopharynx, with variable and non-specific symptoms. Therefore, developing an accurate symptom scale for different regions is essential. Notably, the symptoms of “dryness and burning sensation in the laryngopharynx or mouth” are prevalent among the Chinese population but are often omitted from conventional symptom assessment scales, such as the Reflux Symptom Index (RSI) and Reflux Symptom Score-12 (RSS-12) scales. To address this gap, our study incorporated the symptoms into the RSI and RSS-12 scales, developing the RSI-10/RSS-13 scales. Afterward, we assessed the role of the new scale’s reliability (Cronbach’s α and test–retest reliability), construct validity (confirmatory factor analysis and confirmatory factor analysis), and diagnostic efficiency. Our study encompassed 479 participants (average = 39.5 ± 13.4 years, 242 female) and 91 (average = 34.01 ± 13.50 years, 44 female) completed 24 h MII-pH monitoring. The Cronbach’s α values of 0.80 and 0.82 for the RSI-10 and RSS-13 scales, respectively. RSI-10 and RSS-13 exhibited strong test–retest reliability (ICCs = 0.82–0.96) and diagnostic efficacy (AUC = 0.84–0.85). Furthermore, the factor analysis identified the RSS-13 and its three sub-scales (ear-nose-throat, digestive tract, respiratory tract) exhibited good to excellent structural validity (χ^2^/df = 1.95, *P* < 0.01; CFI = 0.95, RMSEA = 0.06, SRMR = 0.05). The AUC optimal thresholds for the RSI-10 and RSS-13 in the Chinese population were 13 and 36, respectively. Besides, the inclusion of the new item significantly improved the diagnostic efficiency of the RSI scale (*P* = 0.04), suggesting that RSI-10 holds promise as a more effective screening tool for LPRD, and global validation is needed to demonstrate the impact of this new symptom on the diagnosis of LPRD.

## Introduction

Laryngopharyngeal reflux disease (LPRD) is an inflammatory state of the upper respiratory tract tissue correlated with gastroduodenal content reflux, which induces morphologic changes in the upper respiratory tract histology^1^. It is accompanied by non-specific symptoms, such as throat clearing, pain, and hoarseness^2^. A previous study demonstrated that approximately 10.15% of patients in Chinese otorhinolaryngology outpatient clinics had LPRD^3^, and the incidence rate of LPRD has become increasingly higher since changes in diet structure and lifestyle^4^. Statistically, in the USA, the average direct cost for LPRD patients is $5,438, and drug spending is a major part of the first year^5^, which causes significant distress in quality of life (QoL)^6^. It follows that the timely and accurate diagnosis of LPRD is critical. Although 24-h multichannel intraluminal impedance-pH (MII-pH) monitoring is the gold standard for LPRD^7^, it places high demands on healthcare settings. Therefore, screening by symptom scales is still mainstream in clinical practice, especially in rural areas where laryngoscopy is unavailable. Nevertheless, previous studies found that the prevalence of symptoms, scale cutoff, sensitivity, and specificity values varied across areas^8,9^, making the symptom-base scales need to be explored on a region-by-region basis.

A previous study conducted by Lechien JR et al. observed that 53.9% of patients with primary burning mouth syndrome had nonacid or mixed laryngopharyngeal reflux (LPR), and the reflux contents can directly and indirectly damage the laryngopharynx or mouth mucosa^10^. Meanwhile, one study found that dry throat and mouth prevalence in LPRD patients is higher than 80%^11^. Another study by Becker et al.^12^ found burning mouth sensations in 58 of 120 (48.3%) patients with gastroesophageal reflux disease (GERD). However, the tissue susceptibility of the larynx and pharynx compared with the esophagus may explain why some patients experience “dryness and burning sensation in the laryngopharynx or mouth” symptoms of LPR even in the absence of heartburn symptoms^13^. Given that the symptom is lacking in the commonly used scales, such as Reflux Symptom Index (RSI) and Reflux Symptom Score-12 (RSS-12)^14,15^, this would result in underdiagnosis of some patients, and adding the symptom may partially address the diagnostic challenges.

Hence, it is necessary to supplement LPRD symptom-based scales more accurately and systematically across cultures, translate them into different country versions, and apply them to the largest number of LPRD patients. This study aimed to include the symptoms of “dryness and burning sensation in the laryngopharynx or mouth” into the RSI/RSS-12 scales and designed a cross-sectional survey at an otorhinolaryngology clinic in a tertiary hospital to assess the reliability, validity, and diagnostic efficiency alteration between pre- and post-change. Thus, it explores the role of the new symptom in the diagnosis of LPRD and the new scales’ optimal thresholds in the Chinese population.

## Methods

### Patients and design

This study enrolled patients who attended the Ear Nose Throat (ENT) outpatient clinic of Fujian Provincial Hospital from December 2021 to December 2022, using a random selection of patients whose clinic numbers numbered 5. The inclusion criteria were as follows: (1) age ≥ 18 years and (2) ability to accurately cooperate in completing the survey or understanding and following the investigator's instructions. The exclusion criteria were as follows: (1) previous history of throat surgery or laryngeal cancer, vocal cord leukoplakia, and other pharyngeal diseases; (2) alcohol dependence, pregnancy, neurologic or psychiatric illness, and upper respiratory tract infection within the last month and (3) allergies to PPI medications, and history of acid-inhibitory drugs, antibiotics, and hormones within the past four weeks. Questionnaires were distributed to the enrolled patients, and an instructor instructed patients to respond on the spot. The instructor promptly alerted patients to missing data to ensure a comprehensive and accurate response.

**Figure 1:**
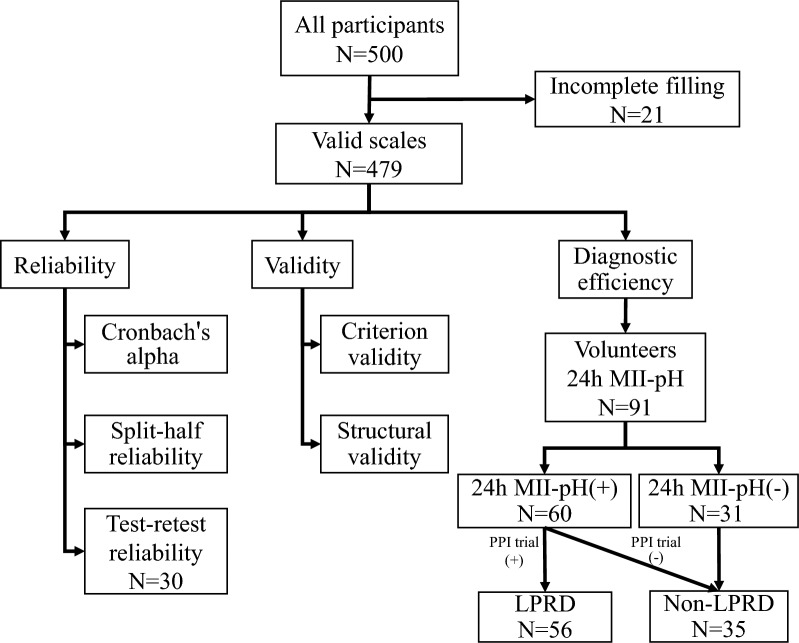
The flow of participants. 24 h MII-pH, 24-h multichannel intraluminal impedance-pH monitoring; PPI, proton-pump inhibitor.

Volunteers were recruited to complete the laryngoscopy examination and 24-h MII-pH monitoring among all outpatients who completed the scale. Eight weeks of proton-pump inhibitor (esomeprazole, 20 mg twice daily) treatment was administered to patients with LPRD according to 24 h MII-pH monitoring results. Follow-ups were conducted after treatment, and the definition as positive for the assignment of LPRD diagnosis was a 50% improvement in the RSI score^16^, as summarized in Fig. 1. Ethical approval was obtained for this study from the Fujian Provincial Hospital Ethics Committee (Ethics Review Approval No: K2021-11-011). All participants signed an informed consent form, and all methods were performed under the relevant guidelines and regulations.

### Questionnaires and sample size calculation

The RSI scale is a 9-item self-reported questionnaire developed to assess the subjective perception of LPRD. Patients indicate severity on a scale of 0 (no symptoms) to 5(very severe)^14^. We introduced the 10th item (dryness and burning sensation in the laryngopharynx or mouth) to RSI and formed the RSI-10 scale. The RSI-10 scores range from 0 to 50.

The RSS-12 scale is a 12-item self-reported questionnaire and includes seven ear, nose, and throat (ENT) items, three digestive items, and two respiratory items. Within the previous month, the frequency, severity, and QoL aspects were evaluated, with frequency ranging from “I don’t have this complaint over the past month” to “complaint occurs daily” and severity ranging from “no symptoms” to “very severe at the time of the attack”. The responses on each aspect ranged from 0 to 5^15^. We introduced the 13th item (dryness and burning sensation in the laryngopharynx or mouth) to RSS-12 scales and formed the RSS-13 scale. The total symptom score is the sum of the "frequency × severity scores" ratings for 13 symptoms, which range from 0 to 325. Meanwhile, the total QoL score ranges from 0 to 65.

Based on the scale principle that the sample size is at least 5 to 10 times that of item^17^, the RSS-13 scale consisted of 13 items, each with three aspects for severity, frequency, and QoL. It was assumed there would be 10–20% invalid questionnaires, so 500 questionnaires were distributed.

### 24 h MII-pH monitoring and laryngoscopy

The 91 volunteers received no food for at least 8 h and water for 4 h before the laryngoscopy and 24 h MII-pH monitoring examination. The 24 h MII-pH monitoring system (Jinshan Science and Technology, Chongqing, China) includes a recorder and monitoring catheter as well as analysis software, which has 4 impedance channels and 1 pH channel. The impedance channels started 0.5 cm above the upper end of the esophageal sphincter (UES) and were arranged 1 cm down sequentially. The pH channel is in the middle of the first two impedance channels. All operations were performed through laryngoscope positioning (Supplementary Fig. 1). The positive diagnostic criteria were as follows^7^: ≥ 1 LPR event or ≥ 5 full column reflux events [reflux 2 cm distal to the upper esophageal sphincter] per day.

### Statistical analysis

The RSI, RSI-10, RSS-12, and RSS-13 scales were evaluated using multiple measures of reliability, validity, and diagnostic efficacy.

### Reliability

The internal consistency reliability was evaluated within each scale using Cronbach's alpha and Spearman-Brown split-half reliability coefficients. Intragroup correlation coefficients (ICCs) were used to demonstrate test–retest reliability through reassessed the 30 patients without treatment after two weeks from the initial assessment. Cronbach's alpha, Spearman-Brown correlations, and ICCs ≥ 0.6 are acceptable, and those ≥ 0.8 are excellent^18–20^.

### Validity

#### Criterion validity

Spearman’s correlation coefficient was used to analyze criterion validity through RSI-10/RSS-13 and RSI/RSS-12, weak (r < 0.3), moderate (0.3 ≦ r < 0.7), or strong (r ≥ 0.7)^21^.

#### Structural validity

To examine the factor structure of the RSS-12 and RSS-13 scales (item > 10), exploratory factor analysis (EFA) and confirmatory factor analysis (CFA) were examined. When Kaiser‒Meyer‒Olkin (KMO) value > 0.6 and Bartlett’s test of sphericity < 0.01 indicates sampling adequacy and suitability for factor analysis^22^. Data were divided into two groups according to the coded singles or doubles: CFA and EFA. In EFA, the factor loadings and cumulative variance contribution rate > 0.4 were considered satisfactory and interpretability^23^. In CFA, the model fit indexes were the comparative fit index (CFI), standardized root mean square residual (SRMR), and root-mean-square error of approximation (RMSEA)^24^. χ^2^/df < 3, RMSEA and SRMR < 0.06 indicate a good fit, > 0.10 indicate an inadequate fit, and recommend that the model be rejected^25^. CFI compares the target model with the original model, with values > 0.90, suggesting an acceptable fit^26^.

### Diagnostic efficiency

The diagnostic ability of all scales were evaluated using the receiver operating characteristic (ROC) analysis, specifically by calculating the area under the curve (AUC) value^27^. A larger AUC indicates a higher accuracy in detecting the disease's presence, and a greater than 0.7 suggests a reasonable estimation. Furthermore, the AUC values were compared using DeLong’s test^28^.

Data were recorded by Epidata (version 3.1) and analyzed using SPSS 24.0 and AMOS 24.0 (SPSS Inc, Chicago, IL). Quantitative data are expressed as the mean ± standard deviation ($\overline{x}$ ± s), and qualitative data are expressed as percentages (%). We performed the Mann‒Whitney U test for the nonparametric data. The chi-square test was used for categorical variable data. All statistical significance levels were considered bilateral, and a *P* < 0.05 indicated the presence of statistically significant differences.

## Results

A total of 500 questionnaires were distributed, removing outlier data, and 479 valid questionnaires were obtained through screening, with an effective response rate of 95.60%. Of these, 237 were males, and 242 were females, ages 18 to 78, with a mean of 39.5 ± 13.4 years. The RSI scale score of 8.16 ± 6.95, RSI-10 scale score of 9.41 ± 7.67, RSS-12 scale symptom score of 29.8 ± 31.5, QoL score of 9.79 ± 10.43, and RSS-13 score of 33.28 ± 34.82, and QoL score of 9.79 ± 10.43 (Table 1).

**Table 1:** The score and correlation analysis of the RSI-10 and RSS-13 scales (N = 479).

(a) Item (RSS-13)	(b) Item (RSI-10)	RSS-13 ($\overline{x}$ ± s)		RSI-10 ($\overline{x}$ ± s)	r-value
		Symptom Score	QoL	Symptom Score	
(1a) Hoarseness or a problem with your voice	(1b) Hoarseness or voice problem	2.03 ± 3.91	0.76 ± 1.09*	0.81 ± 1.18	0.759^†^
(2a) Throat pain or pain during swallowing	/	2.34 ± 4.43	0.87 ± 1.16*	/	/
(3a) Difficulty swallowing (pills, liquids or solid foods)	(4b) Difficulty swallowing food, liquids, or pills	1.66 ± 3.73	0.65 ± 1.06*	0.59 ± 1.09	0.661^†^
(4a) Throat clearing (not cough)	(2b) Clearing your throat	3.64 ± 5.48	1.32 ± 1.33*	1.21 ± 1.43	0.685^†^
(5a) sensation of something being stuck in the throat	(8b) Sensations of something sticking in your throat or a lump in your throat	3.68 ± 5.98	1.25 ± 1.36*	1.46 ± 1.52	0.673^†^
(6a) Excess mucous in the throat and/or post nasal drip sensation	(3b) Excess throat mucus or postnasal drip	3.39 ± 5.27	1.22 ± 1.34*	1.14 ± 1.40	0.685^†^
(7a) Bad breath	/	3.00 ± 4.89	1.14 ± 1.27*	/	/
(8a) Heartburn, stomach acid coming up, regurgitation, burping, or nausea	(9b) Heartburn, chest pain, indigestion, or stomach acid coming up	2.71 ± 4.89	1.06 ± 1.25*	0.86 ± 1.22	0.550^†^
(9a) Abdominal pain or diarrhea	/	1.41 ± 3.21	0.64 ± 1.00*	/	/
(10a) Indigestion, abdominal distension and/or flatus	/	2.14 ± 4.42	0.90 ± 1.15*	/	/
(11a) Coughing (not just throat clearing)	(7b) Troublesome or annoying cough	2.39 ± 4.83	0.94 ± 1.22*	0.84 ± 1.32	0.527^†^
(12a) Breathing difficulties, breathlessness, or wheezing	(6b) Breathing difficulties or choking episodes	1.39 ± 3.35	0.63 ± 0.99*	0.56 ± 1.02	0.650^†^
(13a) dryness and burning sensation in the laryngopharynx or mouth	(10b) dryness and burning sensation in the laryngopharynx or mouth	3.49 ± 5.85	1.27 ± 1.43*	1.25 ± 1.42	0.924^†^
/	(5b) Coughing after you ate or after lying down	/	/	0.70 ± 1.13	/
RSI/RSS-12 total score		29.79 ± 31.48	11.37 ± 8.35*	8.16 ± 6.95	0.768^†^
RSI-10/RSS-13 total score		33.28 ± 34.82	12.64 ± 9.17*	9.41 ± 7.67	0.806^†^

RSI-10, Reflux Symptom Index-10; RSS-13, Reflux Symptom Score-13; RSI, Reflux Symptom Index; RSS-12, Reflux Symptom Score-12; QoL, quality of life; (number and a) indicates the item in the RSS-13 scale; (number and b) indicates the item in the RSI-10; /, Mean no data.

*RSS-13 symptoms score and QoL Spearman’s correlation coefficient, *P* < 0.01.

^†^Two-scale symptoms score Spearman’s correlation coefficient, *P* < 0.01.

### Variation of reliability index

The Cronbach’s alpha coefficients of the RSI, RSI-10, RSS-12, and RSS-13 scales were calculated to be 0.788, 0.800, 0.811, and 0.825, respectively. Meanwhile, the split-half reliability coefficient improved from 0.841 to 0.851 compared to the RSI and RSI-10 scales, while the RSS-12 scale demonstrated an increase from 0.879 to 0.888 upon adding the new item. Additionally, the test–retest reliability assessments conducted on a cohort of 30 patients after a two-week interval yielded coefficients that rose from 0.956 to 0.967 when incorporating the new items in the RSI scale, as Table 2 shows.

**Table 2:** Reliability analysis and criterion validity.

	Cronbach’s alpha	Split-half reliability	Test–retest reliability	RSI^a^	RSS-12^a^
RSI	0.788	0.841	0.956	/	/
RSI-10	0.800	0.851	0.967	0.981	0.790
RSS-12	0.811	0.879	0.817	/	/
RSS-13	0.825	0.888	0.817	0.761	0.987

RSI, Reflux Symptom Index; RSI-10, Reflux Symptom Index-10; RSS-12, Reflux Symptom Score-12; RSS-13, Reflux Symptom Score-13.

^a^According to Spearman’s correlation coefficient.

### Variation of validity index

**Figure 2:**
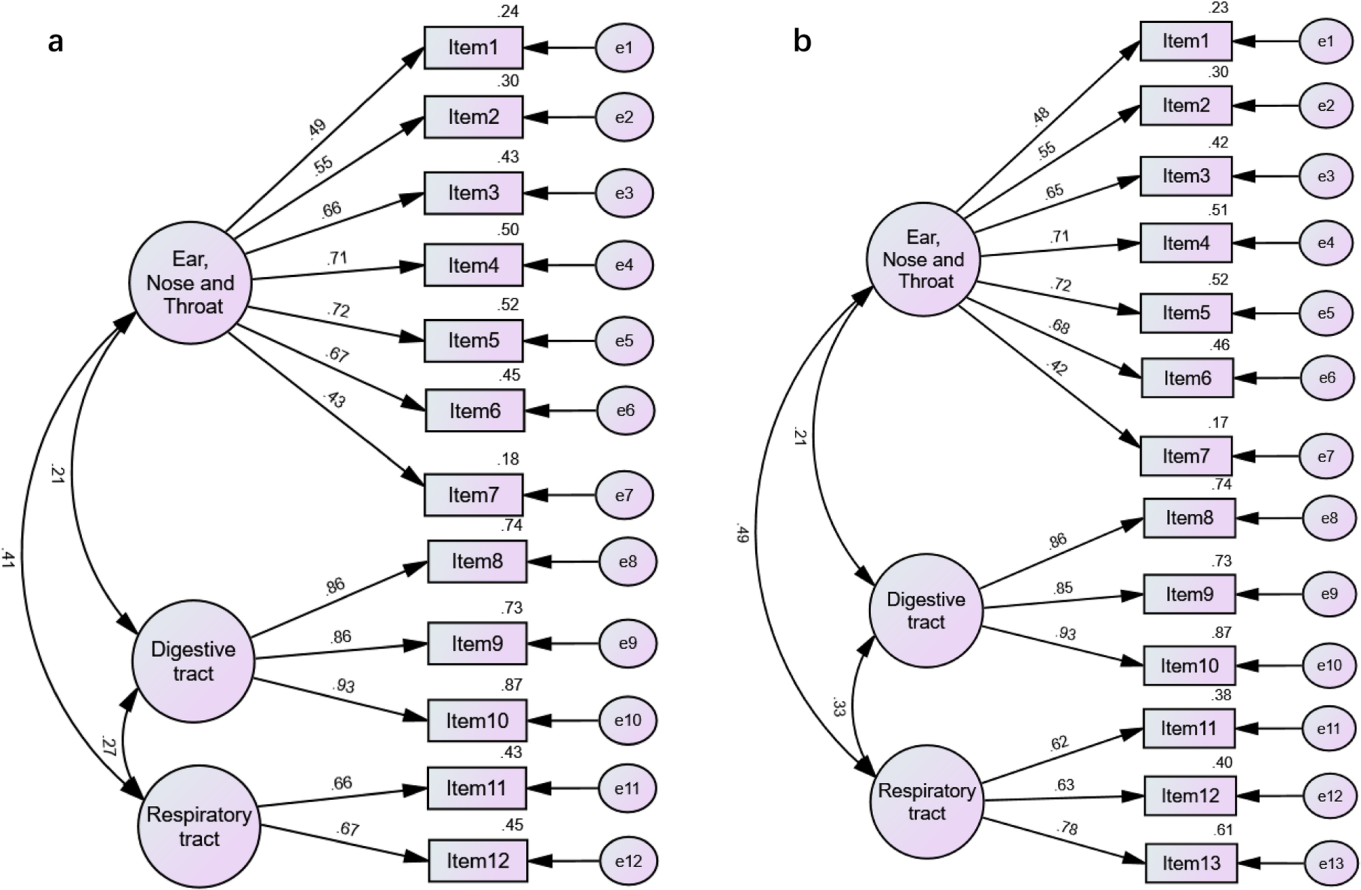
Confirmatory factor analysis (standardized measurement model). (**a**) Reflux symptom score-12(RSS-12), (**b**) RSS-13; the oval and rectangle represent the latent variable and the measured item, respectively; e1 to e13 represent the residual terms of the measure term; the numbers on the arrows are the standardized factor loading values.

The results of the correlation analysis showed strong correlations from RSI to RSI-10 and RSS-12 to RSS-13. The RSS-12 and RSS-13 scales' KMO values are > 0.06, and Bartlett's spherical values are < 0.01. In EFA, the cumulative variance contributions of the RSS-12/RSS-13 scale factors were 61.51% and 59.69%, respectively. Their factor standardized factor loadings ranged from 0.407 to 0.888, and the new item factor loading was 0.568 (Table 3). In CFA, the RSS-12 and RSS-13 standardized regression coefficients of the three factors were 0.42–0.93, all above the standard value of 0.4 (Fig. 2). Furthermore, the RSS-12 scale (χ2/df = 1.94, CFI = 0.95, RMSEA = 0.06, SRMR = 0.05) and RSS-13 scale (χ2/df = 1.95, CFI = 0.95, RMSEA = 0.06, SRMR = 0.05) both exhibited fitness indicators that met or closely approached the model criteria, indicating that the three dimensions are suitable for the diagnosis of LPRD (Supplementary Table 1).

**Table 3:** Exploratory factor analysis (EFA) of RSS-12 and RSS-13 (*N* = 239).

Dimension^a^	Item	RSS-12			RSS-13		
		Factor1	Factor2	Factor3	Factor1	Factor2	Factor3
Ear nose throat (ENT)	(1a) Item	0.407			0.408		
	(2a) Item	0.726			0.728		
	(3a) Item	0.835			0.835		
	(4a) Item	0.739			0.719		
	(5a) Item	0.769			0.765		
	(6a) Item	0.670			0.651		
	(7a) Item		**0.622**			**0.623**	
Digestive tract	(8a) Item		0.802			0.799	
	(9a) Item		0.722			0.720	
	(10a) Item		0.848			0.844	
Respiratory tract	(11a) Item		/	0.879			0.888
	(12a) Item			0.840			0.790
	(13a) Item			/			**0.568**
Characteristics		4.178	1.794	1.408	4.481	1.797	1.482
Cumulative variance contribution		34.820	49.768	61.505	34.468	48.287	59.688

Bold, the item distributed to the dimensions different from the original RSS-12 scale and the new item.

RSS-12, Reflux Symptom Score-12; RSS-13, Reflux Symptom Score-13.

^a^means the dimension in the primary RSS-12 scale.

### Diagnostic efficiency

91 patients participated in the 24 h MII-pH monitoring, with an average age of 34.01 ± 13.50 years, of whom 60 were diagnosed as positive (treated with PPIs for eight weeks), and only 4 had no effect after treatment. The mean age in the LPRD group was 30.54 ± 11.31 years, and 23 (35.0%) were females, with RSI, RSI-10, RSS-12, and RSS-13 on a median of 14, 16, 49, and 55, respectively. Of the 35 non-LPRD patients, have a mean age of 30.5 ± 11.3 years, and 18 (%) were male, with RSI, RSI-10, RSS-12, and RSS-13 on a median of 5, 5, 14, 15, respectively (Table 4). Besides, we found significant improvements in symptom scores after 8 weeks of PPI treatment, and a reduction in the incidence of the dryness/burning sensation from 75.0% to 21.4%.

**Table 4:** Demographic and clinical characteristics of the patients and following-up.

Characteristics	Non-LPRD	LPRD	Following-up
	(n = 35)	(n = 56)	(n = 56)
Gender, n (%)			
Male	17(48.6%)	30(53.6%)	/
Female	18(51.4%)	26(46.4%)	/
Age(years), mean ± SD	30.54 ± 11.31	36.30 ± 14.36	/
Height(cm), mean ± SD	166.17 ± 7.85	167.23 ± 9.76	/
Weight(kg), mean ± SD	64.35 ± 21.00	65.49 ± 14.67	/
BMI (kg/m^2^), mean ± sd	23.31 ± 7.71	22.66 ± 3.46	/
24 h MII-pH, median (IQR)			
LPR events	0 (0, 0)	0.075 (0,1)*	/
Full column reflux	2 (1, 3)	8 (6, 12)*	/
Dryness and burning sensation, n (%)	9 (25.7%)	42 (75%)*	13 (21.4%)^†^
Scale, median (IQR)			
RSI	5 (0, 8)	14 (10, 18.75)*	5 (2, 8)^†^
RSI-10	5 (1, 9)	16 (12.25, 21.75)*	5 (2, 8)^†^
RSS-12	14 (3, 27)	49 (30, 70)*	15 (7, 27)^†^
RSS-13	15 (4, 28)	55.5 (30, 79.75)*	15 (7, 28)^†^

LPRD, Laryngopharyngeal reflux disease; Following-up, LPRD patients were treated with PPI empirically for 8 weeks; 24 h MII-pH, 24-h multichannel intraluminal impedance-pH; RSI, Reflux symptom index; RSI-10, 10-Item RSI; RSS-12, Reflux symptom score-12; RSS-13, Reflux symptom score-13; IQR, Interquartile range.

*mean compare non-LPRD and LPRD groups, *P* < 0.01.

^†^mean compare LPRD and Following-up groups, *P* < 0.01.

**Figure 3:**
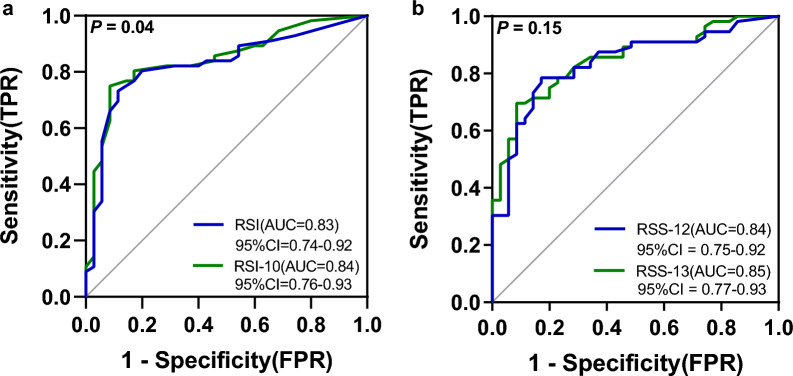
Receiver operating characteristic curve. (**a**) The receiver operating characteristic (ROC) curve of RSI and RSI-10; (**b**) The ROC of RSS-12 and RSS-13; *P* means using DeLong’s test.

The AUC values of RSI, RSI-10, RSS-12, and RSS-13 scales were 0.83 (*sensitivity* 73.2%; *specificity* 88.6%), 0.84 (*sensitivity* 75.0%; *specificity* 91.4%), 0.84 (*sensitivity* 78.6%; *specificity* 82.9%), and 0.85 (*sensitivity* 69.6%; *specificity* 91.4%), respectively (Fig. 3). A statistically significant difference was observed between the RSI and RSI-10 scales, as determined by DeLong's test (*P* = 0.04). The maximum Youden index corresponding values of the RSI, RSI-10 RSS-12, and RSS-13 scales were calculated to be 0.618, 0.664, 0.614, and 0.611, respectively. In addition, the analysis revealed that the optimal cutoff values for these scales were 11, 13, 29, and 36, respectively (Supplementary Table 2).

## Discussion

The symptom of “dryness and burning sensation in the laryngopharynx or mouth” may be a valuable addition to diagnosing LPRD. It ranks among the most common symptoms reported by patients visiting ENT outpatient clinics, and there was a notable decrease in incidence after 8 weeks of PPI treatment. Furthermore, our findings indicated that the RSI and RSS-12 scales exhibit high levels of reliability and validity, and adding the new symptoms enhanced these scales' reliability, validity, and diagnostic efficacy among Chinese populations.

Although the symptom of “Togue burning” was initially included among the items of the RSS scales, it was then eliminated when designing the short version of the instrument (RSS-12) for not reaching a 50% prevalence in European LPRD patients^29^. However, Lechien JR et al.^10^ observed that 53.9% of patients with primary burning mouth syndrome had nonacid or mixed LPR. While, the item of “dryness and burning sensation in the laryngopharynx or mouth” was one of the most frequently reported complaints in our study (75%), and this result aligns with a study conducted by Chen et al.^11^ in which the three most common extraesophageal symptoms in LPRD patients were globus sensation, dry throat/pharyngeal itching, and dry mouth in the Chinese population. The discrepancy between the Chinese and European populations may be due to the following reasons. Firstly, the geographical bias leads to dietary and lifestyle habits playing a vital role in manifesting symptoms. Secondly, the description of “Tongue burning” in the original RSS is inaccurate because the refluxate of LPR first injures laryngopharynx or mouth rather than tongue, and the mucous membranes of the laryngopharynx and mouth, both lateral and posterior, are susceptible to reflux burning than tongue. Therefore, we advocate for further qualitative investigations, aiming to better understand patient experience of dryness/burning.

Consistent with prior studies, the reliability of the RSI and RSS-12 scales was excellent^30–32^. It is worth noting that our research boasts the largest sample size in cross-sectional studies compared to the study mentioned above (479 vs. 273) and explores the diagnostic efficacy of the scale through a longitudinal study. Furthermore, our findings indicate that the inclusion of new items may enhance the RSI/RSS-12 scale's stability, internal consistency, validity and diagnostic efficacy, as demonstrated by multimethod approach, including Cronbach's alpha, Spearman-Brown split-half, test–retest, and factor analysis, etc.

Factor analysis was employed to assess the validity of the RSS-12 and RSS-13 scales. The results of the EFA revealed three factors (ENT, digestive tract, respiratory tract) that were consistent with the original scale^15^, indicating that the RSS-12 scales possessed satisfactory construct validity and that the symptoms of laryngopharyngeal reflux consist of 3 systems are appropriate. However, the seventh item of “bad breath” was distributed to the digestive tract rather than ENT. Some previous studies divided "bad breath" symptoms into oral and nonoral categories, and the pharynx and digestive tract are intimately related to the nonoral tract^33^. A recent study^34^ discovered that methyl mercaptan and hydrogen sulfide concentrations in the pharynx and upper esophagus are significantly higher in patients with pharyngeal reflux than in healthy individuals, which can evaporate “bad breath”. Therefore, “bad breath” items assigned to the digestive tract may be applicable in real life. Similarly, we noticed that “dryness and burning sensation in the laryngopharynx or mouth” was distributed to the respiratory tract. However, the larynx or mouth is anatomically part of the otolaryngology department. The result may also partly conform to reality because the respiratory tract and ENT overlap in the upper respiratory tract. Equivalent to Lechien’s hypothesis^15^, our CFA results showed that the three systems had a good structural validity, which makes us believe that the diagnosis of LPRD needs to be comprehensively evaluated by ENT, digestive tract, and respiratory tract examinations.

Previous research has indicated that the RSI and RSS-12 scales exhibit favorable diagnosability^14,15^. Furthermore, robust criterion validity was observed when comparing these scales with the RSI-10 and RSS-13, as evidenced by a strong correlation (r-value > 0.9, *P* < 0.01), demonstrating that the new symptoms highly agree with the original scale. In addition, we discovered that adding the new item allowed the differentiation of LPRD patients from non-LPRD subjects by plotting ROC curves. The AUC increased significantly between the RSI and RSI-10 scales (*P* < 0.05). Given that the new item is one of the most frequently reported complaints by patients and significant relief after PPI treatment, we concluded that it is an essential supplement for diagnosing LPRD (Fig. 3a). Additionally, Beletsky et al.^12^ discovered that 13 was the optimal RSI scale cutoff point, while in both Kamani^35^ and our studies, the values were 11. Varied diets, geographical circumstances, and even the frequency of symptom manifestation might lead to different results^8,9^. For this reason, future cross-sectional and longitudinal studies based on larger population samples are needed to determine the most appropriate cutoff values and the prevalence of dryness and burning sensations.

The RSS-12 scale encounters a comparable situation. Because the severity score is multiplied by the frequency score to obtain a symptom score ranging from 0 to 25 for each item, the cutoff of 11 in the RSS-12 scale was less than half of the maximum in one item, which might be too low to diagnose LPRD accurately. However, in our investigation, the cutoff value of the RSS-12 scale was 29, meaning that at least two severe symptoms are required to diagnose LPRD, avoiding overdiagnosis, such as due to symptoms of “abdominal pain or diarrhea” caused by gastroenteritis was misdiagnosed as LPRD, which can minimize the waste of resources. Although our RSS-12 scales exhibited a lower AUC, sensitivity, and specificity than the original study^15^, it is suitable for the Chinese population. Additionally, our team discovered that compared with the RSI-10, the RSS-13 took more time to complete, but there were no statistically significant differences in AUC between the RSS-12/RSS-13 and RSI-10 scales. Thus, the RSI-10 might be a more promising screening tool for LPRD, but it still needs translations in multiple cultures, and leveraging scales in e-health/m-health platforms for wider screening access to validity assessments. These additional studies will be instrumental in corroborating our findings and could potentially influence the development of global LPRD management guidelines.

Although the symptom of “dryness and burning sensation in the laryngopharynx or mouth” is so prevalent, there are several limitations. First, as a single-center study, selection bias is possible, and further studies are necessary at the international level (recruiting patients of different nationalities) using standardized protocols to confirm the results. Second, we found that LPRD patients with higher symptom scores may be more likely to participate in 24 h MII-pH monitoring, leading to a positive rate of 61.5%. Third, our study did not give the PPI treatment to patients with 24 h MII-pH negativity, which may lead to a missed diagnosis of false-negative patients.

## Conclusion

Validated and accurate questionnaires are essential for the global study of patients with LPRD. The item “dryness and burning sensation in the laryngopharynx or mouth” is so prevalent that the RSI-10/RSS-13 scales have better reliability, validity, and diagnostic ability for evaluating patients with LPRD in the Chinese population. In addition, the RSI-10 might be a more promising screening scale.

## Supplementary Information

Supplementary Information.

## Data Availability

Individual participant data will not be shared due to ethical restrictions. The anonymized datasets generated and analyzed during the current study are available from the corresponding author upon reasonable request.
