# Author Correction: Assessing the role of dryness and burning sensation in diagnosing laryngopharyngeal reflux

**DOI:** 10.1038/s41598-025-91973-2

**Published:** 2025-03-07

**Authors:** Xiaowei Zheng, Zhiwei Chen, Ting Chen, Liqun Zhou, Chaofeng Liu, Jingyi Zheng, Renyou Hu

**Affiliations:** 1 Department of Otorhinolaryngology Head and Neck Surgery, Shengli Clinical Medical College of Fujian Medical University, 134 Dong Jie, Gulou District, Fuzhou City, 350001 Fujian Province China; 2 Chongqing Jinshan Science and Technology (Group) Co Ltd, Chongqing, 401120 China

Correction to: *Scientific Reports* 10.1038/s41598-024-55420-y, published online 24 February 2024

The Acknowledgements section in the original version of this Article was incomplete. The Acknowledgements section now reads:

“This study was supported by the National Natural Science Foundation of China (81970899); Fujian Province Social Development Guiding (key) Projects (2021Y0048); the Major Scientific Research Program for Young and Middle‐aged Health Professionals of Fujian Province, China, Grant no. 2022ZQNZD001.”

The original Article has been corrected.

