# Supplementary material for: Assessing the role of dryness and burning sensation in diagnosing laryngopharyngeal reflux
Source: Sci Rep. 2024 Feb 24;14:4542. doi: 10.1038/s41598-024-55420-y (PMC10894288; doi:10.1038/s41598-024-55420-y)
Supplement: Supplementary file 1 — Supplementary Information. [file 41598_2024_55420_MOESM1_ESM.pdf]

Supplementary Materials

Supplementary Table 1. Confirmatory factor analysis-fit statistics (N = 240)

Model	χ2	df	χ2/df	*P* value	CFI	RMSEA	SRMR
Criteria			<3		>0.9	<0.06	<0.06
RSS-12	98.759	51	1.936	<0.001	0.954	0.063	0.050
RSS-13	120.669	62	1.946	<0.001	0.949	0.063	0.053

Abbreviations: df, degrees of freedom; CFI, comparative fit index; RMSEA, root mean square error of approximation; SRMR, standardized root mean square residual.

Supplementary Table 2. Diagnostic efficacy of the scales (N = 91)

	RSI	RSI-10	RSS-12	RSS-13
Cutoff	10.5	12.5	28.5	36.0
Sensitivity	0.732	0.750	0.786	0.696
Specificity	0.886	0.914	0.829	0.914
Positive predictive value	0.911	0.933	0.880	0.929
Negative predictive value	0.674	0.696	0.707	0.653
AUC	0.827	0.844	0.836	0.850
95%CI	0.738-0.916	0.761-0.928	0.752-0.921	0.772-0.928

Abbreviations: Abbreviations: RSI, Reflux Symptom Index; RSI-10, Reflux Symptom Index-10; RSS-12, Reflux Symptom Score-12; RSS-13, Reflux Symptom Score-13; AUC, area under the curve.

Supplementary Figure

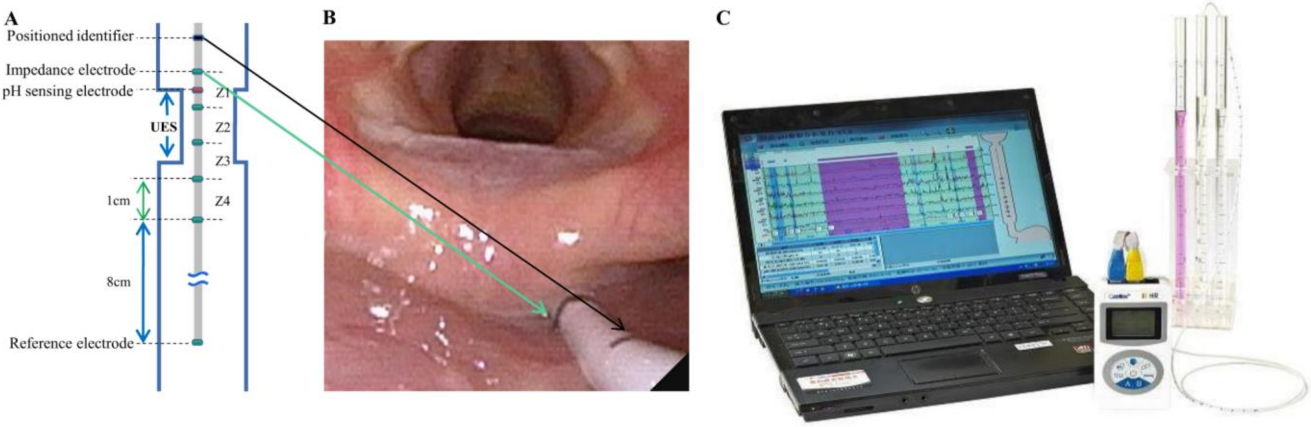

Supplementary Figure 1. The 24h Multichannel impedance-pH (24h MII-pH) monitoring

UES, Upper esophageal sphincter. (A) The 24h MII-pH Catheter internal structure schematic diagram. (B) The 24h MII-pH Catheter localization under laryngoscope. (C) The 24h MII-pH monitoring picture of actual products
